# Arbutin improves gut development and serum lipids *via Lactobacillus intestinalis*

**DOI:** 10.3389/fnut.2022.948573

**Published:** 2022-09-09

**Authors:** Jie Ma, Shuai Chen, Yuying Li, Xin Wu, Zehe Song

**Affiliations:** ^1^Animal Nutritional Genome and Germplasm Innovation Research Center, College of Animal Science and Technology, Hunan Agricultural University, Changsha, China; ^2^Key Laboratory of Systems Health Science of Zhejiang Province, School of Life Sciences, Hangzhou Institute for Advanced Study, University of Chinese Academy of Sciences, Hangzhou, China; ^3^Institute of Bast Fiber Crops, Chinese Academy of Agricultural Sciences, Changsha, China

**Keywords:** arbutin, gut development, gut microbiota, fecal microflora transplantation, *Lactobacillus intestinalis*

## Abstract

Arbutin has been widely studied in whitening, anti-inflammatory, and antioxidant. However, the interaction between arbutin and intestinal microbes has been rarely studied. Thus, mice were treated with arbutin concentrations of 0, 0.1, 0.2, 0.4, and 1 mg/ml. We found that arbutin promoted gut development such as villus length, villus areas, and villus length/crypt depth (L/D). Total cholesterol (TC), high-density lipoprotein (HDL), and low-density lipoprotein (LDL) were significantly reduced by low concentrations of arbutin. Importantly, we analyzed the microbial composition in the control and 0.4 mg/ml arbutin group and found that the abundance of *Lactobacillus intestinalis* (*L. intestinalis*) was highest and enhanced in arbutin. Further, mice were fed with oral antibiotics and antibiotics + 0.4 mg/ml arbutin and then we transplanted fecal microbes from oral 0.4 mg/ml arbutin mice to mice pretreated with antibiotics. Our results showed that arbutin improves gut development, such as villus width, villus length, L/D, and villus areas. In addition, *L. intestinalis* monocolonization was carried out after a week of oral antibiotics and increased villus length, crypt depth, and villus areas. Finally, *in vitro* arbutin and *L. intestinalis* co-culture showed that arbutin promoted the growth and proliferation of *L. intestinalis*. Taken together, our results suggest that arbutin improves gut development and health of *L. intestinalis*. Future studies are needed to explore the function and mechanism of *L. intestinalis* affecting gut development.

## Introduction

Arbutin is a natural phytochemical active substance, which is extracted from the bearberry leaves of *Ericaceae* and *Saxifragaceae* families (1, 2). It inhibits the activity of tyrosinase to reduce the production of melanin in the host (3), thereby lowering the deposition of melanin (4, 5). Meanwhile, arbutin is also associated with antioxidant (6, 7) and anti-inflammatory (8, 9). Additionally, arbutin has been widely studied for its role in protecting against nerve injury or other diseases caused by nerve injury (10, 11). However, arbutin regulation of gut development and host metabolism through gut microbiota has rarely been reported. The intestinal villi were directly contacted with nutrients and absorbed small molecules into the blood (12, 13), whereas the crypt was genetically regulated to shrink and invaginate (14), which was not conducive to nutrient absorption. Goblet cells secrete mucins and mucopolysaccharides to form the mucous system and were the site of colonization by gut microbes (15). Arbutin was rarely absorbed by the small intestine, but the majority was used by gut microbiota. Numerous studies reported that the role of phytochemicals was weakened by low bioavailability (16). Arbutin is a β-glucoside derived from hydroquinone (HQ) (1,4-dihydroxybenzene) (2, 3), its bioactivity and bioavailability were altered by gut microbes secreting glycoside hydrolase (17), and gut microbes have been identified as closely related to host metabolic disorders and diseases (18, 19).

Whether arbutin regulates gut development and host metabolism by altering gut microbes is unclear. Thus, we speculated that the interaction between arbutin and intestinal microbiome influences the pathological status and development of the gastrointestinal tract. Our results indicated that arbutin directly affects the composition of gut microbiota and development; further, *Lactobacillus intestinalis* (*L. intestinalis*) may serve as the potential mechanism.

## Materials and methods

### Bacterial strains

The *L. intestinalis* (ATCC49335) used in this study was purchased by Beijing Beina Chuanglian Biotechnology Research Institute (Beijing, China). Unless otherwise stated, bacterial strains were grown in MRS Broth (MRSB) (Qingdao Hope Bio-technology Corporation Ltd.) or on MRS Agar (MRSA) plates at 37°C.

### Animal studies

Fifty female C57BL/6 mice (aged 6 weeks, 17 ± 0.5 g and aged 4 weeks, 14 ± 0.5 g) were randomly divided into 5 groups with arbutin solution of 0, 0.1, 0.2, 0.4, and 1 mg/ml (20) and fed maintenance diet lasted 3 weeks. We found that arbutin 0.4 mg/ml was most effective in improving intestinal index; thus, twenty mice were treated with antibiotics and antibiotics + 0.4 mg/ml arbutin for 3 weeks. Then, we transplanted fecal microbes from oral 0.4 mg/ml arbutin mice to mice pretreated with antibiotics for 1 week and then normal feeding for 2 weeks. Finally, twenty mice were pretreated with antibiotics for a week, 1.3 × 10^9^ colony-forming unit (CFU)/ml *L. intestinalis* was intragastric to mice for 1 week, and then normal feeding for 2 weeks. All the animals were purchased from Hunan SJA Laboratory Animal Corporation Ltd. (Changsha, China) and used in this study. All the experimental animals were allowed free access to food and drinking water, and subjected to 12-h light-dark cycles, controlled temperature (23 ± 2°C), and humidity (45–60%) during the experiment. The basic diet was described in our previous study (21).

### Hematoxylin and eosin staining

Intestinal HE staining was performed. The jejunal and ileal segments were fixed in 4% paraformaldehyde solution. The sections were first treated with xylene and ethanol solution for 15 and 5 min, respectively, then stained with hematoxylin for 5 min, rinsed with water for 5 min, then stained with eosin solution for 1–3 min, and then washed with ethanol and sealed. Finally, the villi, and crypt morphology were observed under a microscope.

### Serum biochemical parameters

Serum samples were separated after centrifugation at 1,500 × *g* for 10 min at 4°C and 100 μl serum was transferred into another tube. Serum biochemical parameters were determined using an Automatic Biochemistry Analyzer (Cobas c 311, Roche).

### Antibiotic treatment and fecal microflora transplantation

To eradicate commensal bacteria, filter-sterilized drinking water was supplemented with ampicillin (0.5 mg/ml, Meilunbio), gentamicin (0.5 mg/ml, Meilunbio), metronidazole (0.5 mg/ml, Meilunbio), neomycin (0.5 mg/ml, Meilunbio), and vancomycin (0.25 mg/ml, Meilunbio) for 1 week. Antibiotics were purchased from Dalian Meilun Biotechnology Corporation Ltd. (Dalian, China). Before fecal microbiota transplantation, the native gut microbiota in one group of C57 female mice (*n* = 10 biologically independent animals per group) was deleted by administering drinking water containing a cocktail of antibiotics for 1 week. Fecal samples of ∼200 mg were then collected from arbutin (0.4 mg/ml)-fed mice and resuspended in 2.0 ml normal saline. Fecal samples were mixed and centrifuged at 1,000 × *g*, and the microbiota supernatants were transplanted into the microbiota-depleted mice by gavaging with 0.2 ml per mice for 1 week. After transplantation, two groups of mice were administrated with a standard diet and regular water.

### Gut microbiota profiling

Total genome DNA from ileal chyme and mucosa was extracted using cetyltrimethylammonium bromide (CTAB) method. DNA concentration and purity were monitored on 1% agarose gels. According to the concentration, DNA was diluted to 1 ng/μl using sterile water. 16S rDNA genes of distinct regions (16S V3-V4) were amplified using a specific primer (515F-806R) with the barcode. All the PCR reactions were carried out with 15 μl of the Phusion^®^ High-Fidelity PCR Master Mix (New England Biolabs), 2 μM of forward and reverse primers, and about 10 ng of template DNA. Sequencing libraries were generated using the TruSeq^®^ DNA PCR-Free Sample Preparation Kit (Illumina, United States) following the manufacturer’s recommendations and index codes were added according to our previous study (22). Microbial communities were investigated by iTag sequencing of 16S rDNA genes (23, 24).

### Statistical analysis

All the statistical analyses were performed using the one-way ANOVA and *t*-test analysis in SPSS version 20.0 software (SPSS Incorporation, Chicago, IL, United States). The data are expressed as the means ± SEM). *P* < 0.05 was considered statistically significant. All the figures in this study were drawn using GraphPad Prism version 8.0.

## Results

### Arbutin administration improves gut development

**FIGURE 1:**
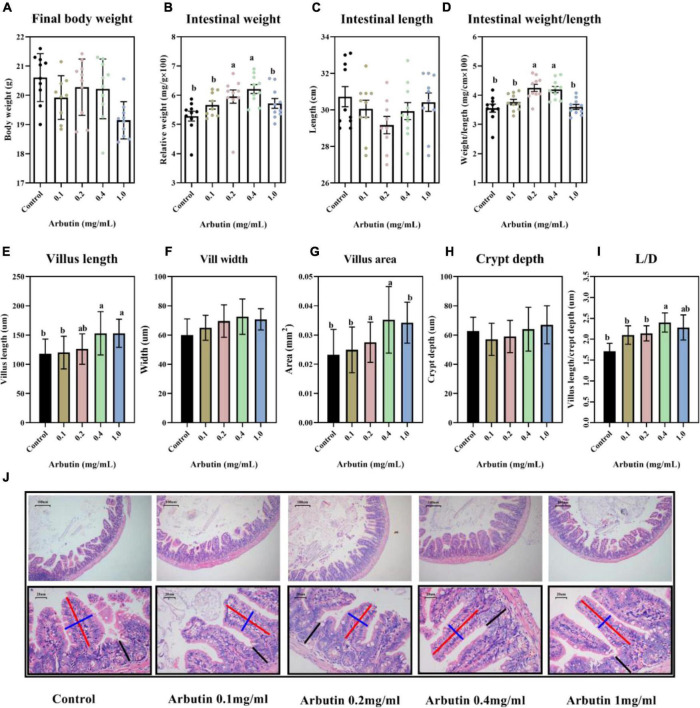
Oral arbutin improves gut development. Final body weight **(A)**, relative intestinal weight **(B)**, intestinal length **(C)**, intestinal weight/length **(D)**, villus length **(E)**, villus width **(F)**, villus areas **(G)**, crypt depth **(H)**, L/D **(I)**, and HE staining of jejunum and ileum **(J)**. Values are presented as the means ± SEMs. Differences were assessed by one-way ANOVA and denoted as follows: a and b indicate significant differences in each group.

Final body weight was not obviously changed (Figure 1A), but the relative weight and weight/length of the intestine were significantly increased by arbutin administration at 0.2 and 0.4 mg/ml (*P* < 0.05) (Figures 1B,D), and arbutin did not alter the intestinal length, villus width and crypt depth (Figures 1C,F,H). Thus, we continued to investigate the intestinal pathology section; the results showed that the villus length was increased by 0.4 and 1.0 mg/ml arbutin, villus area was enhanced by 0.2 and 0.4 mg/ml arbutin, and villus length/crypt depth (L/D) was higher at 0.4 mg/ml arbutin (*P* < 0.05) (Figures 1E,G,I,J).

### Effects of arbutin on serum biochemical parameters

**FIGURE 2:**
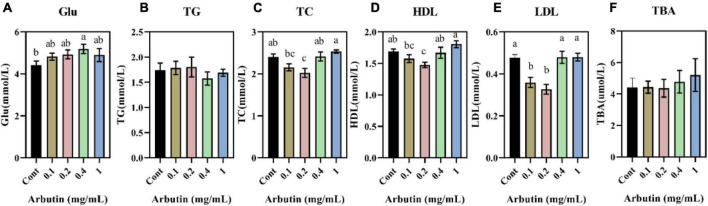
Effects of oral arbutin on serum lipids. Glucose **(A)**, total triglycerides **(B)**, total cholesterol **(C)**, high-density lipoprotein **(D)**, low-density lipoprotein **(E)**, and total bile acid **(F)** (*n* = 10). Values are presented as the means ± SEMs. Differences were assessed by one-way ANOVA and denoted as follows: a and b indicate significant differences in each group.

To further understand the role of arbutin, lipid parameters in serum were determined (Figures 2A–F). Arbutin at 0.4 mg/ml significantly enhanced the content of serum glucose (Glu) (*P* < 0.05) (Figure 2A). Nevertheless, arbutin at 0.2 mg/ml lowered the content of total cholesterol (TC) and high-density lipoprotein (HDL) (*P* < 0.05) (Figures 2C,D), and low-density lipoprotein (LDL) was lowered at 0.1 and 0.2 mg/ml (*P* < 0.05) (Figure 2E). These results suggested that arbutin can improve intestinal development and serum lipid parameters.

### Arbutin alters the composition of gut microbiota

**FIGURE 3:**
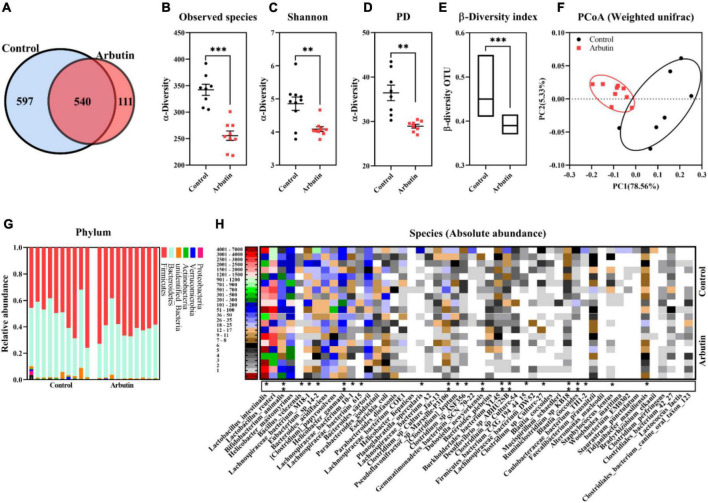
Arbutin alters the gut microbiota. Venn diagram **(A)**, observed species **(B)**, Shannon index **(C)**, PD_whole_tree **(D)**, β-diversity index **(E)**, principal component analysis **(F)**, phylum **(G)**, and species **(H)** were analyzed at 0.4 mg/ml. Differences were assessed by *t*-test and denoted as follows: *P < 0.05, ** *P* < 0.01, ***P < 0.001.

**FIGURE 4:**
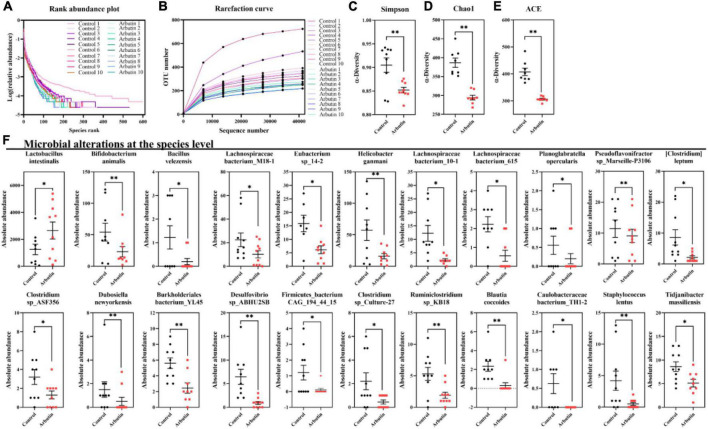
Arbutin alters the gut microbiota. Rank abundance plot **(A)**, rarefaction curve **(B)**, Simpson index **(C)**, Chao1 **(D)**, ACE **(E)**, and species **(F)** were analyzed at 0.4 mg/ml. Differences were assessed by *t*-test and denoted as follows: *P < 0.05, **P < 0.01.

To investigate the effects of arbutin on gut microbes, we determined the microbiome by 16S rDNA sequencing at 0.4 mg/ml (Figures 3, 4). Venn diagram showed that 597 and 111 different operational taxonomic units (OTUs) were found in the control and arbutin groups and contained the same 540 OTUs (Figure 3A), rarefaction curve indicated that the sample capacity and sample depth were reasonable (Figure 4B). Arbutin significantly decreased the α-diversity index [observed species, Shannon index, phylogenetic diversity (PD), Simpson index, Chao1, and abundance-based coverage estimator (ACE)] (*P* < 0.05) (Figures 3B–D, 4A,C–E). Meanwhile, the β-diversity index was reduced (*P* < 0.05) (Figure 3E), and principal component analysis showed that there were different zones of intestinal microflora between the control group and arbutin (Figure 3F). At the phylum level, the relative abundance of *Actinobacteria* and *Proteobacteria* was clearly lowered by arbutin (*P* < 0.05) (Figure 3G). At the species level, 0.4 mg/ml arbutin markedly increased the abundance of *Lactobacillus intestinalis* (*P* < 0.05) (Figures 3H, 4F), while the abundance of *Bifidobacterium animalis*, *Bacillus velezensis*, *Lachnospiraceae bacterium_M18-1*, *Eubacterium* sp_*14-2*, *Helicobacter ganmani*, *Lachnospiraceae bacterium_10-1*, *Lachnospiraceae bacterium_615*, *Planoglabratella opercularis*, *Pseudoflavonifractor* sp_*Marseille-P3106*, *Clostridium leptum*, *Clostridium* sp_*ASF356*, *Dubosiella newyorkensis*, *Burkholderiales bacterium_YL145*, *Desulfovibrio* sp_*ABHU2SB*, *Firmicutes_bacterium CAG_194_44_15*, *Clostridium* sp_*Culture-27*, and *Ruminiclostridium* sp_*KB18* was lowered compared to control (*P* < 0.05) (Figures 3H, 4F).

### Arbutin improves gut development with an antibiotics cocktail and fecal microflora transplantation

**FIGURE 5:**
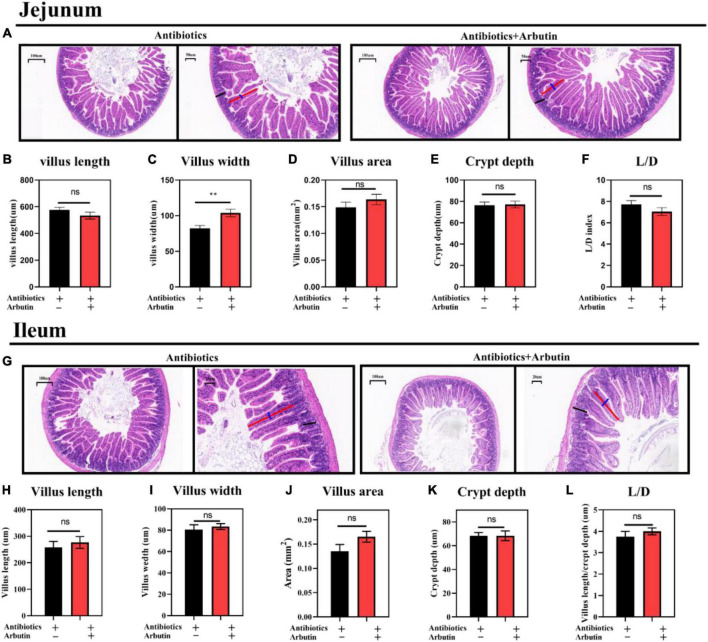
Arbutin administration improved jejunal and ileal gut development after oral cocktails. HE staining of the jejunum **(A)**, villus length in the jejunum **(B)**, villus width in the jejunum **(C)**, villus areas in the jejunum **(D)**, crypt depth in the jejunum **(E)**, L/D in the jejunum **(F)**, HE staining of the ileum **(G)**, villus length in the ileum **(H)**, villus width in the ileum **(I)**, villus areas in the ileum **(J)**, crypt depth in the ileum **(K)**, and L/D in the ileum **(L)** (*n* = 10). Differences were assessed by *t*-test and denoted as follows: **P < 0.01.

**FIGURE 6:**
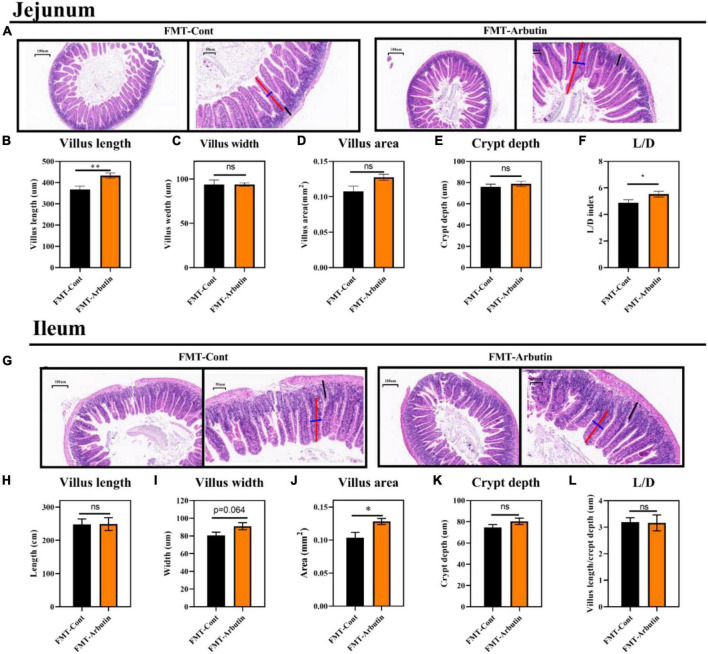
Fecal microflora transplantation improved gut development in mice. HE staining of the jejunum **(A)**, villus length in the jejunum **(B)**, villus width in the jejunum **(C)**, villus areas in the jejunum **(D)**, crypt depth in the jejunum **(E)**, L/D in the jejunum **(F)**, HE staining of the ileum **(G)**, villus length in the ileum **(H)**, villus width in the ileum **(I)**, villus areas in the ileum **(J)**, crypt depth in the ileum **(K)**, and L/D in the ileum **(L)** (*n* = 10). Differences were assessed by *t*-test and denoted as follows: *P < 0.05, **P < 0.01.

The intestinal microbiota has been shown to regulate intestinal development (25) and host metabolism (18). To further determine the role of intestinal microbiota, 4 weeks mice were given an antibiotics cocktail for 1 week with oral arbutin solution (0.4 mg/ml). Predictably, arbutin significantly enriched the villi width compared to the antibiotics group in the jejunum (*P* < 0.05) (Figures 5A,C), but villus length, villus area, crypt depth, L/D were not changed (Figure 5B,D–F), and there was a tendency to enhance the ileal villi index (Figures 5G–L). Then, we further collected feces from mice administered with arbutin 0.4 mg/ml and transplanted them to mice with an antibiotics cocktail. Fecal microflora transplantation significantly improved intestinal pathologies, such as jejunal villus length (Figures 6A,B), jejunal villus length/villus width (L/D) (Figures 6A,F), and ieal villus areas (Figure 6J). But jejunal villus width, jejunal villus area, jejunal crypt depth, ileal villus legth, ileal villus width, ileal crypt depth and ileal L/D were uninfluential (Figures 6C–E,H,I,K,L). In summary, gut microbes contributed improving intestinal development.

### *Lactobacillus intestinalis* colonization reduces gut damage after an antibiotics cocktail

**FIGURE 7:**
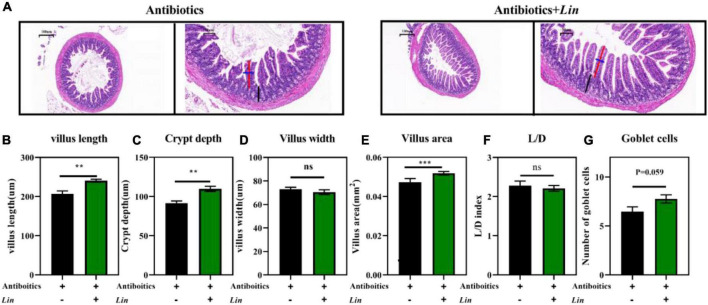
*Lactobacillus intestinalis* colonization improved intestinal development in mice. HE staining the ileum **(A)**, villus length in the ileum **(B)**, villus width in the ileum **(C)**, villus areas in the ileum **(D)**, crypt depth in the ileum **(E)**, L/D in the ileum **(F)**, and the number of goblet cells **(G)** (*n* = 10). Differences were assessed by *t*-test and denoted as follows: **P < 0.01, ***P < 0.001.

We have found that the abundance of *L. intestinalis (Lin)* was markedly enhanced by arbutin and was the most abundant bacterium in the gut (Figures 3H, 4F). *L. intestinalis* was often found in the gut of the host, which was treated for various diseases (26–28) and metabolic disorders (29, 30), but the effect of *L. intestinalis* on gut development and host lipid metabolism was unclear. Thus, we investigated the role of *Lin* on intestinal pathology and used *Lin* monocolonization (31) with an antibiotics cocktail for 1 week. Interestingly, after an antibiotics cocktail for 1 week, *Lin* monocolonization clearly increased the villus length, crypt depth, and villus areas (Figures 7A–C,E), and there was a tendency to elevate the number of goblet cells (Figures 7A,G). Whereas villus width and L/D were not changed by *L. intestinalis* (Figures 7D,F).

### Arbutin promotes the growth of *Lactobacillus intestinalis in vitro*

**FIGURE 8:**
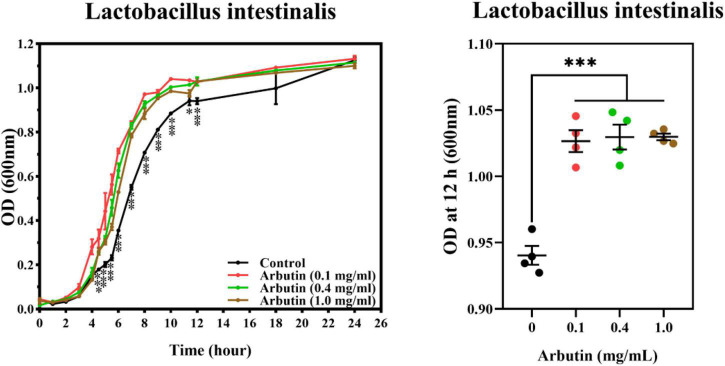
Arbutin promotes the growth of *Lactobacillus intestinalis in vitro*. Arbutin and *L. intestinalis* were co-cultured and determined the OD value at 0–24 h. Differences were assessed by *t*-test and denoted as follows: *P < 0.05, ***P < 0.001.

In order to verify the previous results, we co-cultured arbutin and *L. intestinalis* to investigate the growth of *L. intestinalis in vitro*. The results showed that arbutin significantly promotes the growth of *L. intestinalis* (*P* < 0.05) (Figure 8).

## Discussion

In recent years, arbutin has often been extensively studied that it inhibited tyrosinase activity to reduce melanin deposition in the cosmetic industry (32). Meanwhile, arbutin is often used to treat various diseases, such as types of cancers, central nervous system disorders, osteoporosis, diabetes, and so on (20). Arbutin, as a phytochemical active substance, is low bioavailability in the gut, and incompletely played a beneficial role (33). Further, they are degraded by microbes to increase their biological activity (34). However, the potential of arbutin has rarely been reported about promoting intestinal health. Thus, we explored the effects of arbutin on gut health in the common condition, oral antibiotic cocktails, fecal microflora transplantation, and *Lin* monocolonization.

We investigated the effects of different concentrations of arbutin on gut health and serum lipids in mice in normal conditions. We found that low concentrations of arbutin reduced serum lipids, whereas reversed at high concentrations. Previous studies have shown that arbutin significantly reduced adipocyte differentiation and promoted fatty acid uptake in 3T3-L1 adipocytes (35). The polyjuice decoction containing arbutin decreased the total cholesterol, triglyceride, VLDL, and LDL in diabetic rats (36). Importantly, we found that arbutin plays an important role in promoting intestinal development. In this trial, 0.4 and 1.0 mg/kg arbutin markedly enhanced the villus length, villus areas, and L/D compared to control. Villus index was highly associated with nutrient absorption and gut health (37); thus, the increased villus length, villus areas, and L/D indicated a positive role of arbutin in gut nutrient absorption. The gut microbiota may be an important reason for this result. Arbutin, as a natural phytochemical, is a β-glucoside derived from hydroquinone (2, 3) whose bioactivity and bioavailability can be modified by glycoside hydrolase activity of gut microbiota through the release of acylglycines (38). Microorganisms are associated with the absorption and metabolism of arbutin, a novel *Janthinobacterium* strain (SNU WT3), isolated from the kidney of rainbow trout showed that different biochemical details such as arbutin compared to its close relatives identified (39). Further, *Bifidobacterium* was proved to degrade arbutin (containing glycosides) to elevate bioavailability by secreting β-glucosidase (38). We found that *L. intestinalis* was significantly increased by arbutin, which played an important role in gut health and metabolic disorders (26, 29, 40). However, the abundance of another 21 species of bacteria (such as *Bifidobacterium animalis*, *Bacillus velezensis*, *Lachnospiraceae bacterium_M18-1*, *Eubacterium* sp_*14-2*, and *Helicobacter ganmani*) was significantly reduced. Interestingly, arbutin was reported to reduce colitis symptoms and inhibit lipopolysaccharide-induced inflammation (41), and there were significant negative correlations between arbutin contents and the enriched gut microbiota (e.g., *Eubacterium* and *Ruminococcus*) (42), suggesting that there was bactericidal ability about arbutin.

Gut damage is often associated with drugs, environmental stress, and lifestyle (43). Especially, antibiotics are considered only beneficial, but also potentially harmful drugs, as their abuse appears to play a role in the pathogenesis of several disorders associated with microbiota impairment (44). In this trial, we demonstrated the beneficial effects of arbutin in improving gut health with antibiotics cocktail and fecal microflora transplantation. The result was attributed to arbutin administration altering the gut microflora, such as *L. intestinalis*. Fecal microflora transplantation is a common technique for the treatment of host metabolic disorders and diseases (45, 46). The gut microbiota development of cesarean section infants was rapidly restored by orally derived fecal microflora transplantation (47). Fecal microflora transplantation played beneficial effects on gastrointestinal transport and intestinal barrier dysfunction (48), which were related to intestinal permeability and pathology (49), such as villus length, villus areas, and L/D. Furthermore, the monocolonization technique improves the gut microbiota structure and metabolic process of the host (50, 51) and is also one of the measures to investigate bacterial function. For example, probiotic colonization improved intestinal barrier function and intestinal health, newly identified health-associated bacteria, such as *Faecalibacterium prausnitzii*, *Akkermansia muciniphila*, *Ruminococcus bromii*, and *Roseburia* species (52, 53). Our results showed that *L. intestinalis* monocolonization reduced intestinal damage after an antibiotics cocktail, such as villus length, crypt depth, villus areas, and the number of goblet cells.

To prove the effect of arbutin on *L. intestinalis* growth, we used the co-culture method of arbutin and *L. intestinalis*. Previous studies have found that Bifidobacterium degraded β-glucosidase to enhance the activity of glycoside by secreting β-glucosidase (38). Liu et al. identified a glycoside hydrolase, which is very important for the growth of type I rhamnogalacturonan acid by commensal bacteroides (54). We found that arbutin significantly promoted the proliferation of *L. intestinalis*, suggesting the potential of arbutin on *L. intestinalis* proliferation and utilizing arbutin to increase biological activity.

## Conclusion

Arbutin, as a phytochemical, has been widely studied in whitening, anti-inflammatory, and antioxidant, while the interaction between arbutin and intestinal microbes has been rarely studied. In this trial, we focused on the effects of arbutin on intestinal development and microbes. Predictably, arbutin played a positive role in the gut, such as improving the pathological state of the jejunum and ileum and altering the intestinal microbial structure. In addition, we demonstrated the beneficial effects of arbutin on intestinal development through fecal microflora transplantation and *L. intestinalis* monocolonization by antibiotic cocktail therapy. However, the specific mechanisms of *L. intestinalis* in intestinal development need to be further explored.

## Data availability statement

The datasets presented in this study can be found in online repositories. The names of the repository/repositories and accession number(s) can be found in the article/supplementary material. The data presented in the study are deposited in the NCBI (https://dataview.ncbi.nlm.nih.gov/object/PRJNA839245) repository, accession number: PRJNA839245.

## Ethics statement

The animal model and experimental procedures used in this experiment were approved by the Hunan Agricultural University Institutional Animal Care and Use Committee (202005).

## Author contributions

JM was the primary investigator in this study. SC participated in the animal experiments. YL performed the statistical data analysis. XW participated in the sample analysis. ZS examined the manuscript. All authors contributed to the article and approved the submitted version.
